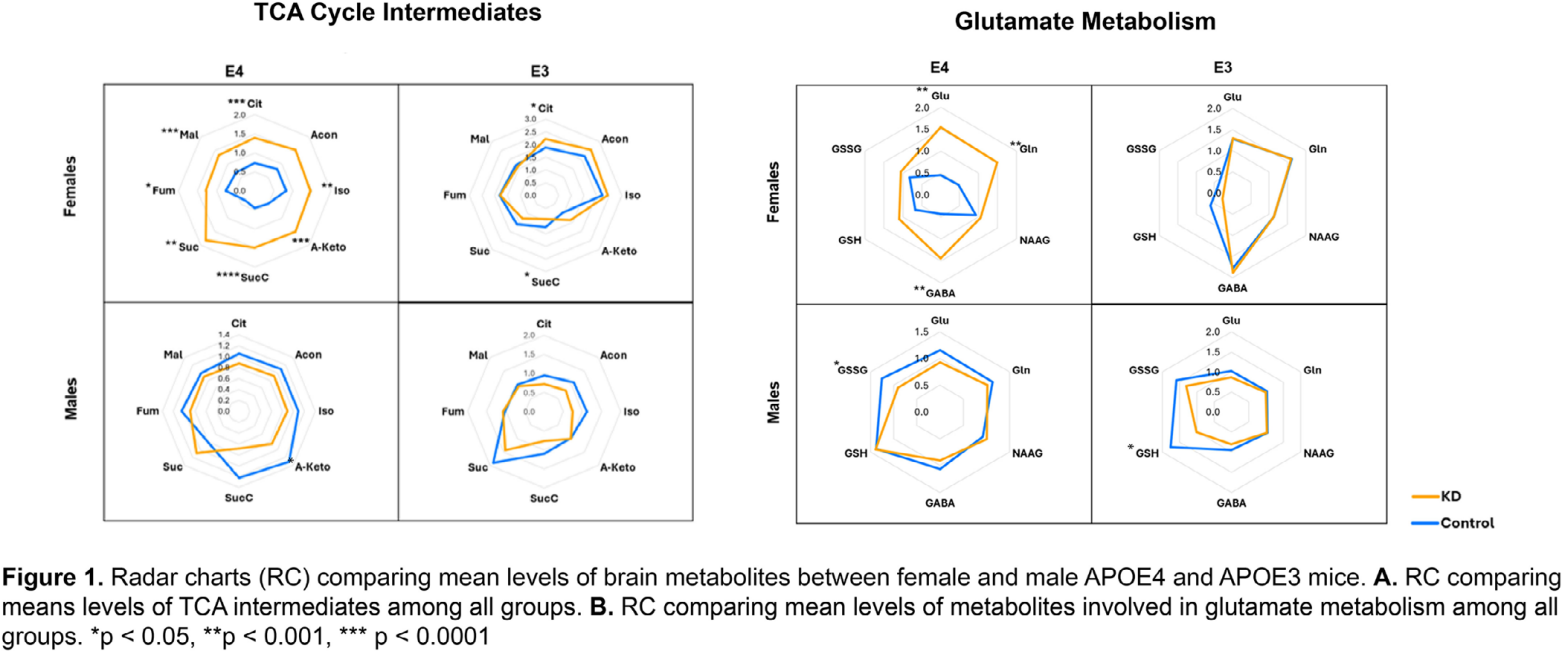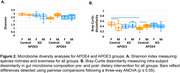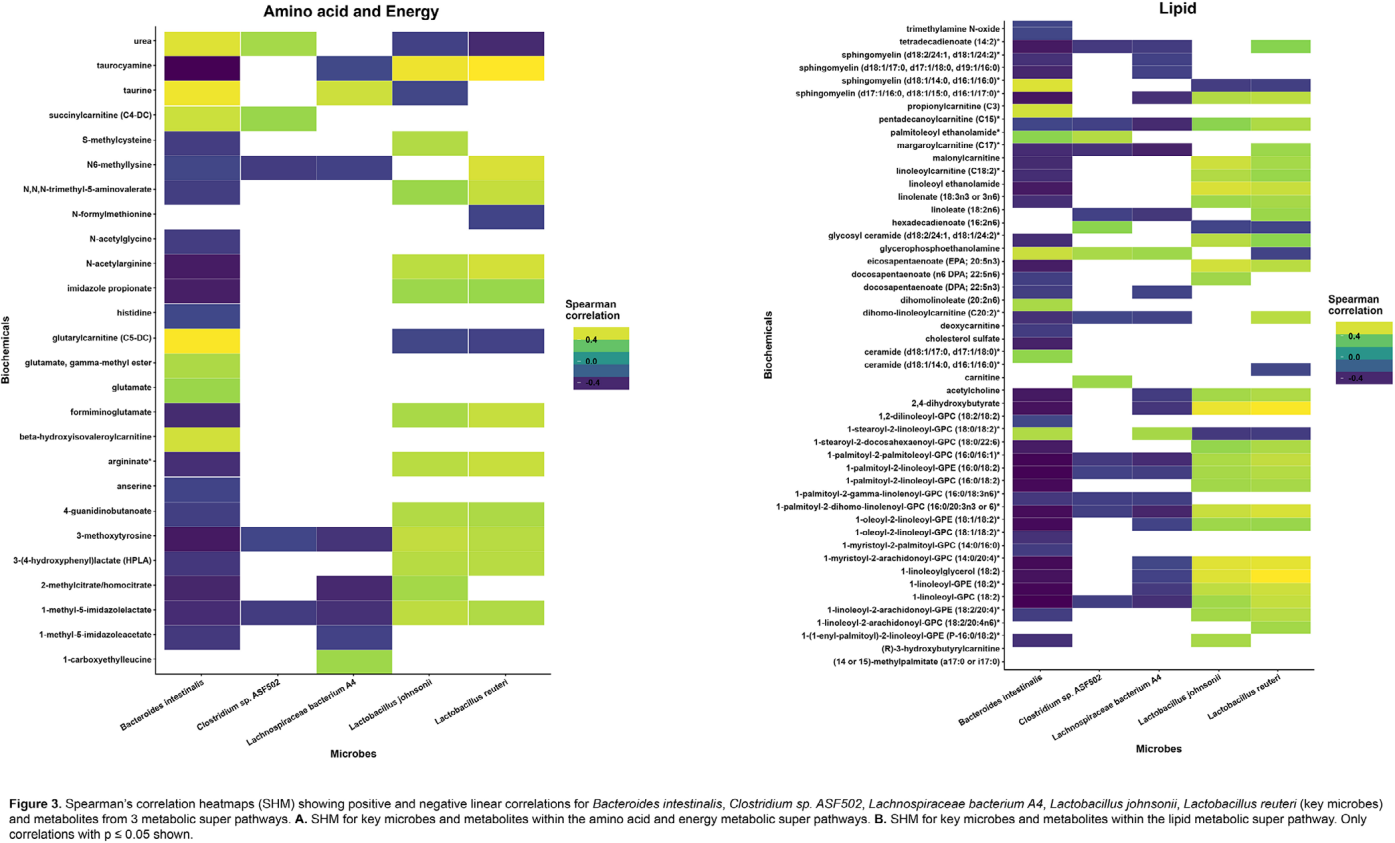# The Ketogenic Diet Restores Brain Metabolism and Gut Microbiome Composition in Female APOE4 Transgenic Mice: A Multiomics Approach

**DOI:** 10.1002/alz70855_104646

**Published:** 2025-12-24

**Authors:** Kira Ivanich, Andrew Yackzan, Ya‐Hsuan Chang, Chetan Aware, Maalavika Govindarajan, Skyler Kramer, Lucille M Yanckello, Aaron Ericsson, Ai‐Ling Lin

**Affiliations:** ^1^ University of Missouri, Columbia, MO, USA; ^2^ University of Kentucky, Lexington, KY, USA

## Abstract

**Background:**

The apolipoprotein ε4 (APOE4) polymorphism is the primary genetic risk factor for Alzheimer's disease (AD). APOE4 carriers exhibit early deficits in brain metabolism and gut microbiome diversity, both elevating AD risk. This study investigated whether a ketogenic diet (KD) can restore brain metabolism and gut microbiome diversity in young, asymptomatic APOE4‐positive mice, while also assessing sex‐based differences, given the higher AD risk in females. Comparisons were also made with APOE3 mice, which carries a neutral AD risk, to determine genotype differences. Additionally, a correlative analysis explored relationships between microbes and brain metabolites, identifying potential therapeutic and screening targets for AD risk mitigation.

**Method:**

Female and male APOE3 (*n* =  44) and APOE4 (*n* =  39) transgenic mice were randomly assigned to a control diet (5.1% fat) or a KD (75.1% fat). Mice ate *ad libitum* for 16 weeks, starting at 12 weeks of age. Brain tissue was collected for untargeted metabolomics (UPLC‐MS/MS via Metabolon Inc.), and fecal samples were collected for 16s rRNA shotgun metagenomic sequencing (CosmosID). Gut microbiome species richness and evenness were measured using Shannon index (α‐diversity). Bray‐Curtis dissimilarity (β‐diversity) measured intra‐subject dissimilarity for pre‐ and post‐diet gut microbiome composition, and Spearman's correlation heatmaps linked metabolites and microbes to correlations within amino acid, energy, and lipid metabolic pathways.

**Result:**

The KD restored brain metabolism in APOE4 females by recovering levels of metabolites associated with mitochondrial function (Figure 1A) and glutamate metabolism (Figure 1B), while exerting variable effects on these metabolites in APOE3 mice and APOE4 males. The KD increased species’ richness and evenness in APOE4 females (Figure 2A) and balanced microbiome composition in APOE4 mice, as indicated by limited changes pre‐ and post‐dietary intervention (Figure 2B). Correlation analyses revealed that *Bacteroides intestinalis, Clostridium sp. ASF502, Lachnospiraceae bacterium A4, Lactobacillus johnsonii, Lactobacillus reuteri* had significant associations with metabolites involved in amino acids and energy (Figure 3A) and lipid (Figure 3B) pathways.

**Conclusion:**

The KD effectively restored brain metabolism and gut microbiome diversity in APOE4 female mice. These effects were absent in APOE3 mice and APOE4 males. Correlations between microbes and metabolites provide potential targets for AD interventions and risk assessment.